# Low‐Dose Oral Versus Topical Minoxidil in Androgenetic Alopecia: A Randomized, Double‐Blind, Double‐Dummy Controlled Trial

**DOI:** 10.1111/jocd.71198

**Published:** 2026-09-21

**Authors:** Muhammad Asad Ejaz Chaudhry, Bushra Muneeb, Safoora Aamir, Zoha Arif Saeed, Sidra Ashraf, Shoaib Ashraf

**Affiliations:** ^1^ Department of Dermatology Shaikh Zayed Hospital Lahore Pakistan; ^2^ Department of Radiology Vancouver General Hospital Vancouver Canada; ^3^ Department of Biochemistry College of Veterinary Sciences Jhang Pakistan; ^4^ Department of Biomedical Sciences Ross University School of Veterinary Medicine Basseterre Saint Kitts and Nevis

**Keywords:** androgenetic alopecia, hypertrichosis, low‐dose oral minoxidil, minoxidil, pattern hair loss, randomized controlled trial, South Asian

## Abstract

**Background:**

Androgenetic alopecia (AGA) is a prevalent, progressive condition with significant psychosocial impact. Current pharmacological modalities like topical minoxidil and finasteride have limitations. Low‐dose oral minoxidil has surfaced as a valid off‐label solution, with the practical advantage of once‐daily dosing.

**Objective:**

To evaluate the efficacy and safety of topical and oral minoxidil in AGA.

**Methods:**

In this double‐blind, double‐dummy randomized controlled trial, 100 patients aged 18–50 years with AGA were randomized to receive either twice‐daily topical minoxidil (5% in males, 2% in females) plus a placebo tablet, or once‐daily oral minoxidil (2.5 mg in males, 1.25 mg in females) plus a placebo spray for 6 months. The primary outcome was the proportion of patients achieving ≥ +1 improvement in a validated 7‐point global photographic scale. Secondary outcomes included patient satisfaction and adverse effects. Data were analyzed using SPSS v26 with *p* ≤ 0.05 as significant.

**Results:**

Ninety patients completed 24 weeks of follow‐up. Photographic assessment demonstrated that mild‐to‐excellent improvement (≥ +1) occurred in both the oral (67%) and topical minoxidil (73%) groups (*p* = 0.652). Patient‐reported satisfaction was high in both groups (topical: 70.5%; oral: 80.4%, *p* = 0.332), with a non‐significant trend favoring oral minoxidil at the moderate satisfaction threshold (58.7% vs. 38.6%, *p* = 0.057). Median improvement scores were comparable between groups for both clinician assessment and patient‐reported outcomes. Hypertrichosis was significantly more frequent with oral minoxidil (30% vs. 9%, *p* = 0.011), whereas contact dermatitis was exclusive to topical minoxidil (11% vs. 0%, *p* = 0.025). No serious adverse events were observed.

**Conclusion:**

Both topical and low‐dose oral minoxidil demonstrated clinically meaningful improvement in androgenetic alopecia. Despite oral therapy exhibiting a trend toward higher patient satisfaction, this study was not powered to establish non‐inferiority or equivalence. The dermatologist‐assessed outcomes indicated no significant intergroup differences. Low‐dose oral minoxidil may be considered a practical alternative in selected patients, particularly where adherence to topical therapy is limited.

**Trial Registration:**

ClinicalTrials.gov: NCT07273799

## 1 Introduction

Androgenetic alopecia (AGA), also known as pattern hair loss, is the most common cause of hair loss. It affects over 75% of men by age 80, and 50% of women by age 70 [1, 2]. This multifactorial condition, primarily driven by genetic predisposition and androgen excess, leads to the miniaturization of androgen‐sensitive hair follicles by dihydrotestosterone (DHT) [2]. This results in progressive hair loss in the frontal, temporal, and vertex regions of the scalp in males, and the central scalp in females, which is measured clinically by the Norwood‐Hamilton scale in males [1, 3] and Sinclair scale in females [1, 4]. Currently, the only treatments for AGA approved by the FDA are topical minoxidil and oral finasteride.

Minoxidil has multiple mechanisms of action for promoting hair growth. Primarily, it acts as a potent vasodilator, enhancing blood flow to hair follicles via vascular endothelial growth factor and hypoxia‐inducible factor 1A [5]. Additionally, it prolongs the anagen phase by promoting prostaglandin synthesis, which influences WNT/beta‐catenin activity in dermal papilla cells and facilitates conversion of vellus hair to terminal hair. Lastly, minoxidil causes the opening of ATP‐sensitive K channels important for cell proliferation [6, 7].

Non‐compliance with topical minoxidil is a significant challenge for dermatologists due to several factors. It has limited efficacy (up to 60% of patients), and visible hair growth takes 4 months. An initial phase of shedding may occur in the first 4 to 6 weeks. Only 5% of topical minoxidil applied to the skin is absorbed, with 75% of it during the first 4 h [8]. Patients are therefore advised to avoid shampooing or swimming for at least 4 h following application. Discontinuation of minoxidil typically results in reversal of hair growth gains within 4 months.

Recently, low‐dose oral minoxidil, typically defined as < 5 mg/day, has been increasingly used off‐label to manage various forms of hair loss [9]. The sulfotransferase enzyme, found in epidermal keratinocytes, hair follicles, liver, and platelets, converts minoxidil into its active form, minoxidil sulphate. Hence, it is thought that systemic administration can increase bioavailability and facilitate greater drug accumulation within hair follicles [10, 11]. Other advantages of oral minoxidil that improve patient compliance include ease of use, faster administration, once‐daily dosing, no effect on hairstyle, and the ability to bypass limitations of cutaneous absorption and adverse effects [8]. Despite the high prevalence of AGA in South Asian populations, randomized controlled trials in this population remain limited, particularly those directly comparing oral and topical minoxidil. Accordingly, this study aimed to compare their relative outcomes in terms of safety and efficacy using sex‐specific dosing in this critically underrepresented cohort in the existing literature.

## 2 Methods

This double‐blind, double‐dummy randomized controlled trial (RCT) was conducted at a tertiary care dermatology center, from December 2025 to May 2026. Ethical approval was obtained from the Institutional Review Board, and written informed consent was obtained from all participants before enrollment. The study was conducted in accordance with the principles of the Declaration of Helsinki and reported in line with the Consolidated Standards of Reporting Trials (CONSORT) guidelines. The trial was registered at ClinicalTrials.gov (Identifier: NCT07273799) on December 10, 2025.

Patients aged 18–50 years with clinically diagnosed androgenetic alopecia (AGA) were screened for eligibility. Inclusion criteria comprised Norwood–Hamilton Grade II–VI in males or Sinclair Grade II–V in females, with a minimum duration of hair loss of 1 year and no recent treatment. Patients were excluded if they had a history of cardiovascular disease, prior use of hair restoration therapies (including minoxidil, finasteride, or hair transplantation) within the preceding 6 months, known hypersensitivity to study medications, or were pregnant or lactating. At baseline, all participants underwent a detailed clinical evaluation, including demographic profiling, duration of hair loss, prior treatment history, and comorbidities. Scalp examination and hair pull testing were performed to exclude other causes of alopecia. Disease severity was graded using the Norwood–Hamilton scale for males and the Sinclair scale for females. Baseline laboratory investigations included complete blood count, serum ferritin, and vitamin D levels. Vital parameters, including blood pressure, heart rate, and body weight, were recorded.

A total sample size of 100 participants (50 per group) was calculated based on prior randomized data suggesting a 20%–25% difference in improvement rates between treatment modalities. Assuming a 20% absolute difference, a two‐sided alpha level of 0.05, and 80% statistical power, the calculated sample size was deemed sufficient to detect a clinically meaningful difference between groups.

**FIGURE 1:**
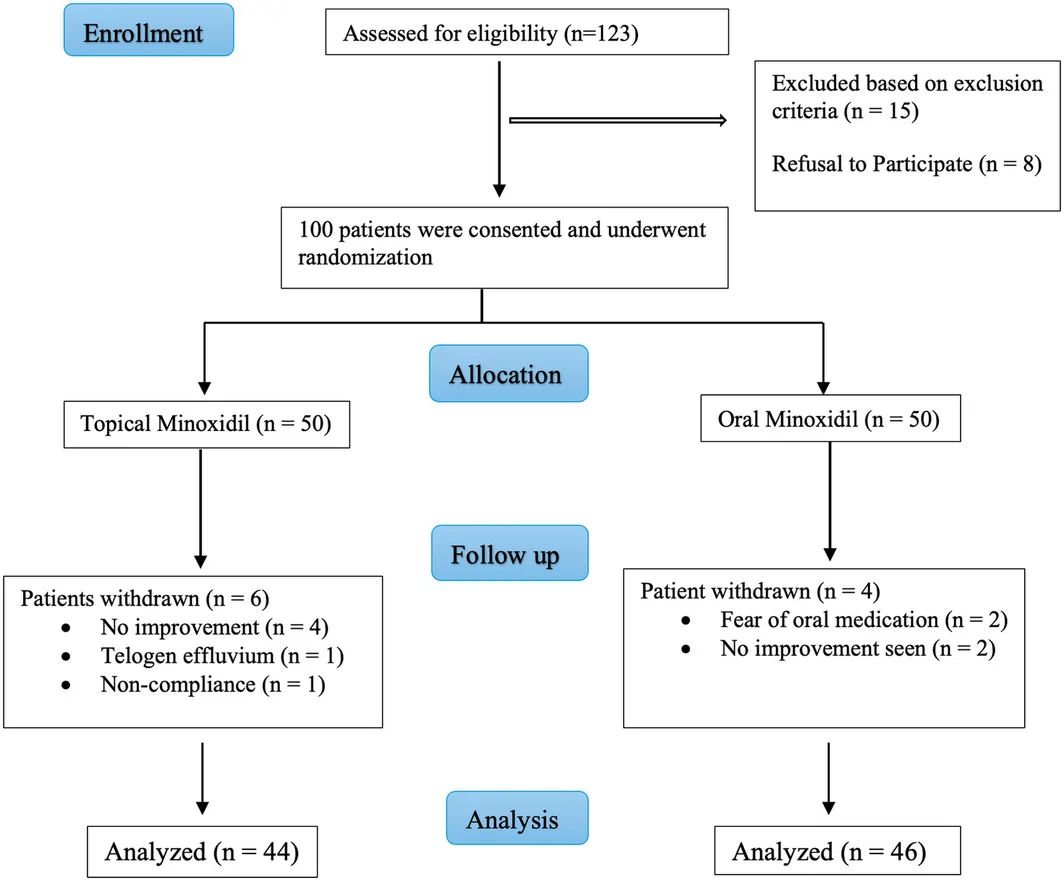
CONSORT flow diagram illustrating the screening, randomization, and allocation of participants.

Participants were randomly assigned in a 1:1 ratio to receive either topical minoxidil with placebo tablets or oral minoxidil with placebo spray. Randomization was performed using a computer‐generated block randomization sequence prepared by an independent investigator not involved in patient recruitment or outcome assessment. Allocation concealment was ensured through the use of sequentially numbered, opaque, sealed envelopes. A double‐dummy design was employed to maintain blinding, whereby all participants received both a topical preparation and an oral tablet. Group A received active topical minoxidil (5% solution in males and 2% in females, 1 mL per application) along with a placebo tablet, while Group B received oral minoxidil (2.5 mg once daily in males and 1.25 mg once daily in females) along with a placebo topical spray. Participants were instructed to apply the topical solution to affected scalp areas twice daily and to take the oral medication at night. Patients, treating clinicians, and outcome assessors were blinded to treatment allocation throughout the study period. Figure 1 displays the CONSORT flow diagram, which illustrates the screening, randomization, and allocation of participants.

Participants were followed up at 4, 12, and 24 weeks over the total treatment duration. At each visit, compliance was assessed through return of used medication containers and patient reporting. Standardized digital photographs were obtained at baseline and at the 24‐week follow‐up visit from frontal, temporal, and vertex views using a consistent imaging protocol. Two independent dermatologists, blinded to treatment allocation, evaluated the images using a validated 7‐point global photographic assessment scale, shown in Table 1, ranging from −3 (severe worsening) to +3 (excellent improvement). Inter‐rater agreement was assessed using Spearman's rank correlation coefficient, which indicated a strong and significant degree of concordance (*r*_s_ = 0.77, *p* < 0.001).

**TABLE 1:** Dermatologist Assessment Scores (DAS) based on 7‐point global photographic assessment scale.

Score		Definition
−3	Severe worsening	1 step downgrade on Norwood scale
−2	Moderate worsening	Clearly visible worsening of hair loss
−1	Mild worsening	Slight increase in hair loss from baseline but not clearly visible
0	No change	No change
1	Mild improvement	Minimal recovery of hairs with bald areas still clearly visible
2	Moderate improvement	Good recovery but not equivalent to uninvolved areas
3	Excellent improvement	Recovery similar to normal areas of scalp

The primary outcome was the proportion of patients achieving at least a +1 improvement from baseline on the global photographic assessment scale at 24 weeks. Secondary outcomes included patient‐reported satisfaction, assessed using a 7‐point Likert scale ranging from −3 (very dissatisfied) to +3 (very satisfied), and the incidence of adverse effects. Safety assessments were conducted through patient‐maintained diaries and clinical evaluation, with specific monitoring for hypertrichosis, dermatologic reactions, orthostatic hypotension, tachycardia, pedal edema, and other systemic symptoms. Blood pressure and heart rate were measured at each visit in both seated and standing positions.

Data were analyzed using the Statistical Package for the Social Sciences (SPSS) version 26 (IBM Corp., Armonk, NY, USA). Normality of distribution was assessed using the Shapiro–Wilk test, and non‐parametric methods were used due to deviation from normality. Continuous variables were summarized as medians with interquartile ranges, and categorical variables as frequencies and percentages. Between‐group comparisons were performed using the Mann–Whitney *U*‐test for continuous variables and the Chi‐squared test or Fisher's exact test for categorical variables, as appropriate. Effect sizes were expressed as absolute risk differences with corresponding 95% confidence intervals. Within‐group changes in Dermatologist Assessment Scores and Patient Satisfaction Scores were evaluated using the Wilcoxon signed‐rank test by comparing median change scores to zero (no change); this analysis was considered exploratory and not intended for between‐group inference. All analyses were conducted only for participants who completed the 24‐week follow‐up. Missing data were not imputed. A *p*‐value of ≤ 0.05 was considered statistically significant.

## 3 Results

Of the enrolled 100 patients with androgenetic alopecia, 90 completed the 24‐week follow‐up and were ultimately included in the primary analysis. In the oral minoxidil group, 4 (8%) dropped out; in the topical group, 6 (12%) discontinued treatment; reasons for discontinuation were similar across groups and detailed in Figure 1. Compliance was high in both groups, with > 90% of empty pill packs and spray bottles returned at each visit.

The cohort comprised 54 men (60%) and 36 women (40%), with a mean age of 30.3 ± 9.1 years (range, 18–50 years). There were no significant differences between the two groups in terms of age, gender, family history, alopecia grade or baseline heart rate (*p* > 0.05), indicating that the two groups were statistically similar at baseline. Table 2 displays the grade of alopecia at the time of enrollment. The majority of male patients were at Stage 2 and 3 (according to Norwood–Hamilton classification), while most female patients were at Stage 2 and 3 according to Sinclair classification.

**TABLE 2:** Baseline characteristics and alopecia grade.

Variable	Topical minoxidil (*n* = 44)		Oral minoxidil (*n* = 46)		*p*
Baseline characteristics					
Sex, *n* (%)^a^					
Female	16 (36.4)		20 (43.5)		0.491
Male	28 (63.6)		26 (56.5)		
Family history of AGA, *n* (%)	19 (43.2)		13 (28.3)		0.140
History of seborrheic dermatitis, *n* (%)	12 (27.3)		11 (23.9)		0.715
Alopecia grade, *n* (%)^a^	Female	Male	Female	Male	
Stage 2	8 (50.0)	6 (21.4)	7 (35.0)	5 (19.2)	—
Stage 3	4 (25.0)	12 (42.9)	6 (30.0)	10 (38.5)	
Stage 4	2 (12.5)	5 (17.9)	4 (20.0)	7 (26.9)	
Stage 5	2 (12.5)	4 (14.3)	3 (15.0)	2 (7.7)	
Stage 6	—	1 (3.6)	—	2 (7.7)	
Total	16 (100)	28 (100)	20 (100)	26 (100)	

^a^ Female patients graded by the Sinclair scale; male patients graded by the Norwood–Hamilton scale.

**FIGURE 2:**
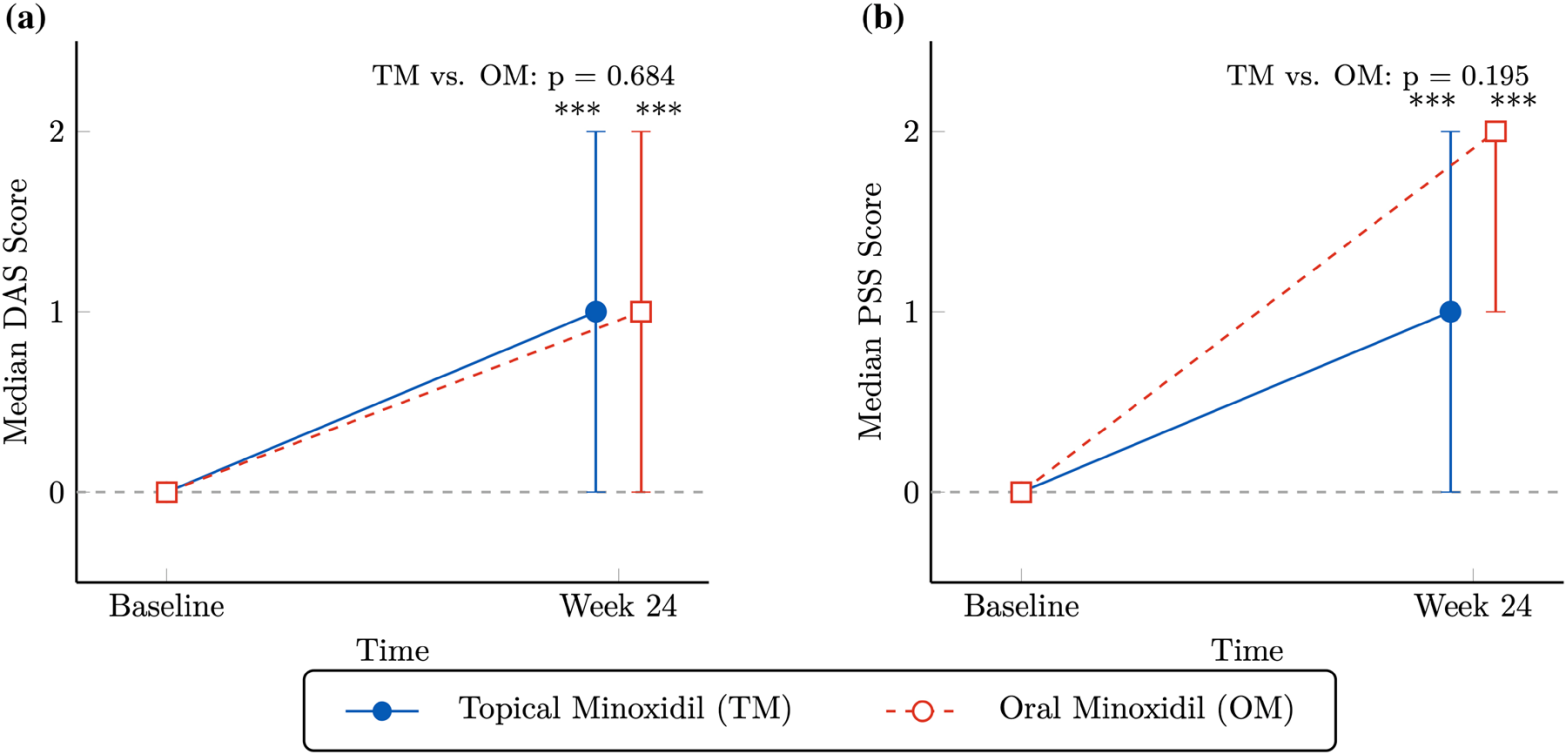
Change in median score from baseline at 24 weeks for (a) Dermatologist Assessment Score (DAS) and (b) Patient Satisfaction Score (PSS). Within‐group efficacy, ***p < 0.001, Wilcoxon signed‐rank test. No significant between‐group difference was detected for DAS (*p* = 0.684) or PSS (*p* = 0.195) (Mann–Whitney *U*‐test). Error bars represent the interquartile range (IQR).

Median Dermatologist Assessment Scores were identical in both groups (median 1.0, IQR 0–2), with a Hodges–Lehmann median difference of 0.0 (95% CI: −1.0 to +1.0; *p* = 0.684), further supporting comparable clinician‐assessed outcomes (Table 3, Figure 2A). In comparison, median Patient Satisfaction Scores were 2.0 (IQR 1–2) in the oral group and 1.0 (IQR 0–2) in the topical group, corresponding to a median difference of +1.0 (95% CI: 0.0 to +1.0; *p* = 0.195) (Table 3, Figure 2B). Although point estimates favored oral therapy, the confidence intervals included no difference, indicating a lack of statistical significance.

**TABLE 3:** Primary and secondary outcomes.

	Group		*p*
	Topical	Oral	
	Median (p2–p75)	Median (p25–p75)	
Age	28.0 (23.0–37.0)	28.0 (23.0–37.0)	0.852
Hair loss grade (male)	3.0 (2.5–3.5)	3.0 (3.0–4.0)	0.258
Hair loss grade (female)	2.5 (2.0–3.5)	3.0 (2.0–4.0)	
DAS at 24 weeks	1.0 (0.0–2.0)	1.0 (0.0–2.0)	0.684
PSS at 24 weeks	1.0 (0.0–2.0)	2 (1.0–2.0)	0.195
Heart rate baseline	74.0 (72.0–76.0)	75.0 (71.0–81.0)	0.127
Heart rate at 24 weeks (HR24)	73.5 (72.0–77.0)	74.0 (71.0–78.0)	0.903
*Δ* systolic BP (mmHg) at 24	0.0 (−6.3 to 8.5)	−1.5 (−8.5 to 5.0)	0.669
*Δ* in diastolic BP (mmHg)	2.0 (−6.0 to 7.0)	0.5 (−5.0 to 8.0)	0.948

*Note:* *Δ* = change at 24 weeks versus baseline.

Abbreviations: DAS, Dermatologist Assessment Score; PSS, Patient Satisfaction Score.

**FIGURE 3:**
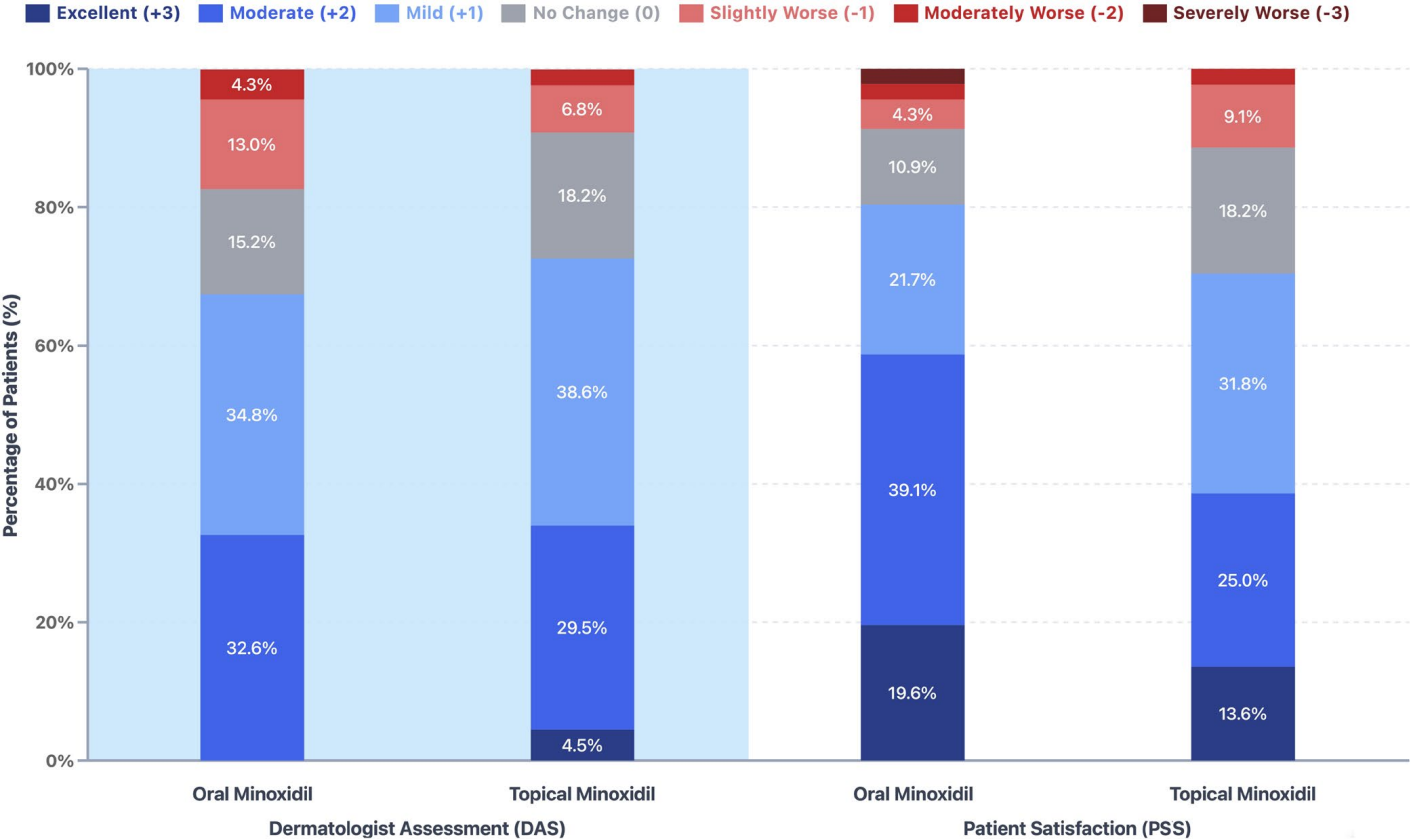
Score distributions at 24 weeks. Dermatologist Assessment Score (DAS) and Patient Satisfaction Score (PSS) assessed on a 7‐point scale ranging from −3 (severe worsening) to +3 (excellent improvement).

Analysis of score distributions demonstrated that most patients in both groups experienced mild‐to‐moderate improvement (+1 to +2). A greater proportion of patients in the oral group achieved higher satisfaction scores (+2 and +3), while worsening scores were uncommon in both groups (Figure 3).

**FIGURE 4:**
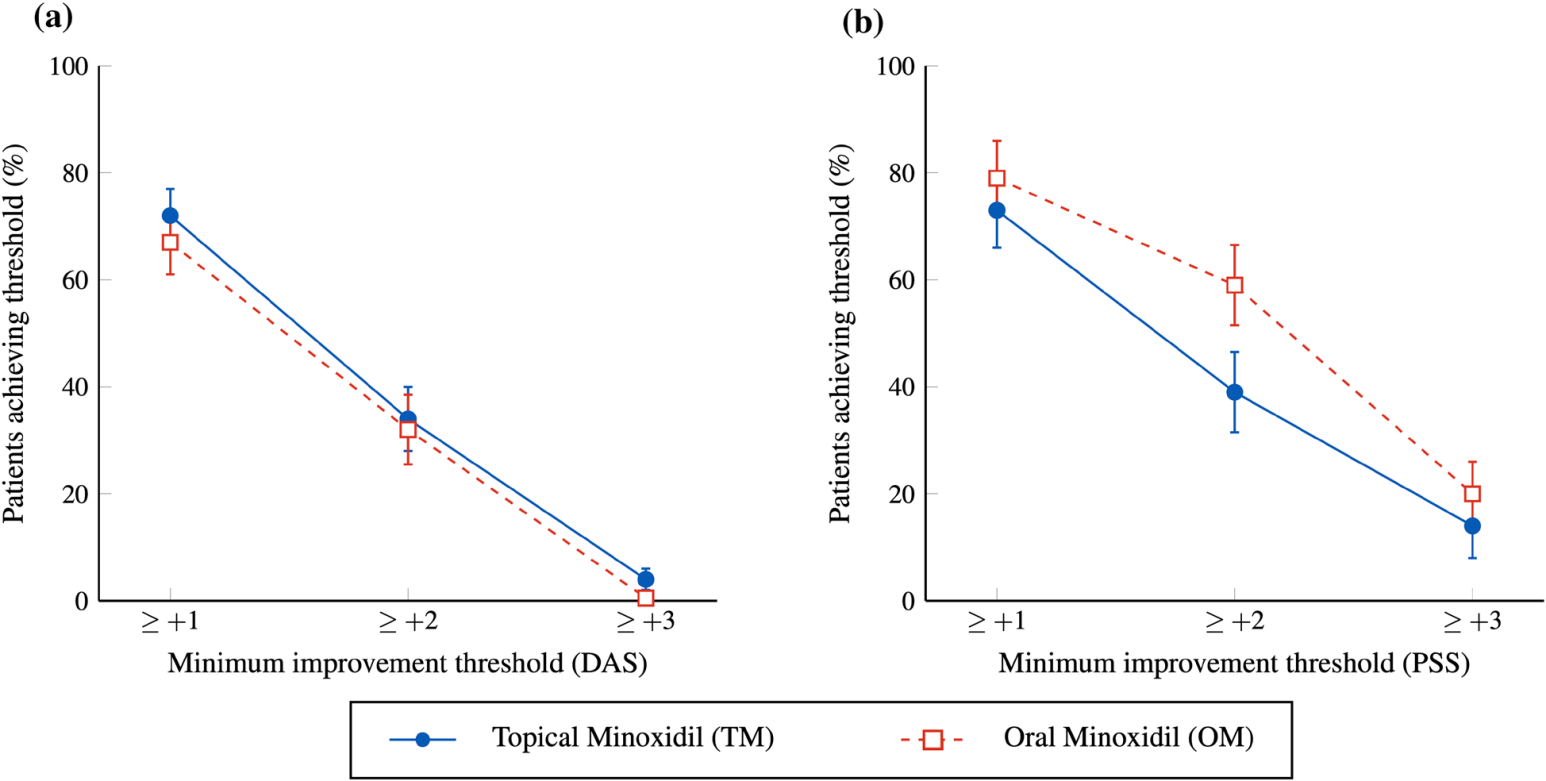
Cumulative responder analysis. Proportion of patients achieving minimum improvement thresholds of ≥ +1, ≥ +2, and ≥ +3 on the (a) Dermatologist Assessment Score (DAS) and (b) Patient Satisfaction Score (PSS) at 24 weeks.

Cumulative responder analysis across increasing thresholds is represented in Table 4 and Figure 4. At 24 weeks, clinician‐assessed improvement (≥ +1 on the global photographic assessment scale) was observed in 67.4% in the oral minoxidil group and 72.7% in the topical group. The absolute risk difference was −5.3% (95% CI: −22.0% to +12.0%; *p* = 0.652), indicating no statistically significant difference between treatments. At higher thresholds (≥ +2 and ≥ +3), response rates declined in both groups and remained comparable. Patient‐reported satisfaction at 24 weeks was high in both groups, with ≥ +1 improvement observed in 80.4% of patients receiving oral minoxidil and 70.5% receiving topical therapy. The absolute risk difference was +9.9% (95% CI: −6.5% to +26.3%; *p* = 0.332). The response rates were consistently higher in the oral group across all thresholds, including ≥ +2 improvement (58.7% vs. 38.6%; risk difference +20.1%, 95% CI: −1.5% to +41.7%, *p* = 0.057), although these differences did not reach statistical significance (Table 4 and Figure 4).

**TABLE 4:** Clinical outcomes with effect sizes and confidence intervals.

Outcome	Topical (%)	Oral (%)	Risk difference (95% CI)	*p*
DAS ≥ +1	72.7%	67.4%	−5.3% (−22% to +12%)	0.652
DAS ≥ +2	34.1%	32.6%	−1.5% (−18% to +15%)	0.883
DAS ≥ +3	4.5%	0%	−4.5% (−12% to +3%)	0.236
PSS ≥ +1	70.5%	80.4%	+9.9% (−6.5% to +26.3%)	0.332
PSS ≥ +2	38.6%	58.7%	+20.1% (−1.5% to +41.7%)	0.057
PSS ≥ +3	13.6%	19.6%	+6.0% (−10.0% to +22.0%)	0.451

*Note:* Risk difference calculated as Oral − Topical. Positive values favor oral minoxidil.

Within‐group analysis demonstrated significant improvement from baseline in both Dermatologist Assessment Scores and Patient Satisfaction Scores at 24 weeks in each treatment arm (*p* < 0.001 for all comparisons; Table 5). These findings confirm that both interventions were associated with measurable improvement over the study period but do not inform comparative efficacy between groups.

**TABLE 5:** Within‐group efficacy analysis.

Outcome	Group	*Z*‐value	*p*
DAS at 24 weeks vs. baseline	Topical	−4.29	< 0.001
	Oral	−3.61	< 0.001
PSS at 24 weeks vs. baseline	Topical	−4.24	< 0.001
	Oral	−4.64	< 0.001

**FIGURE 5:**
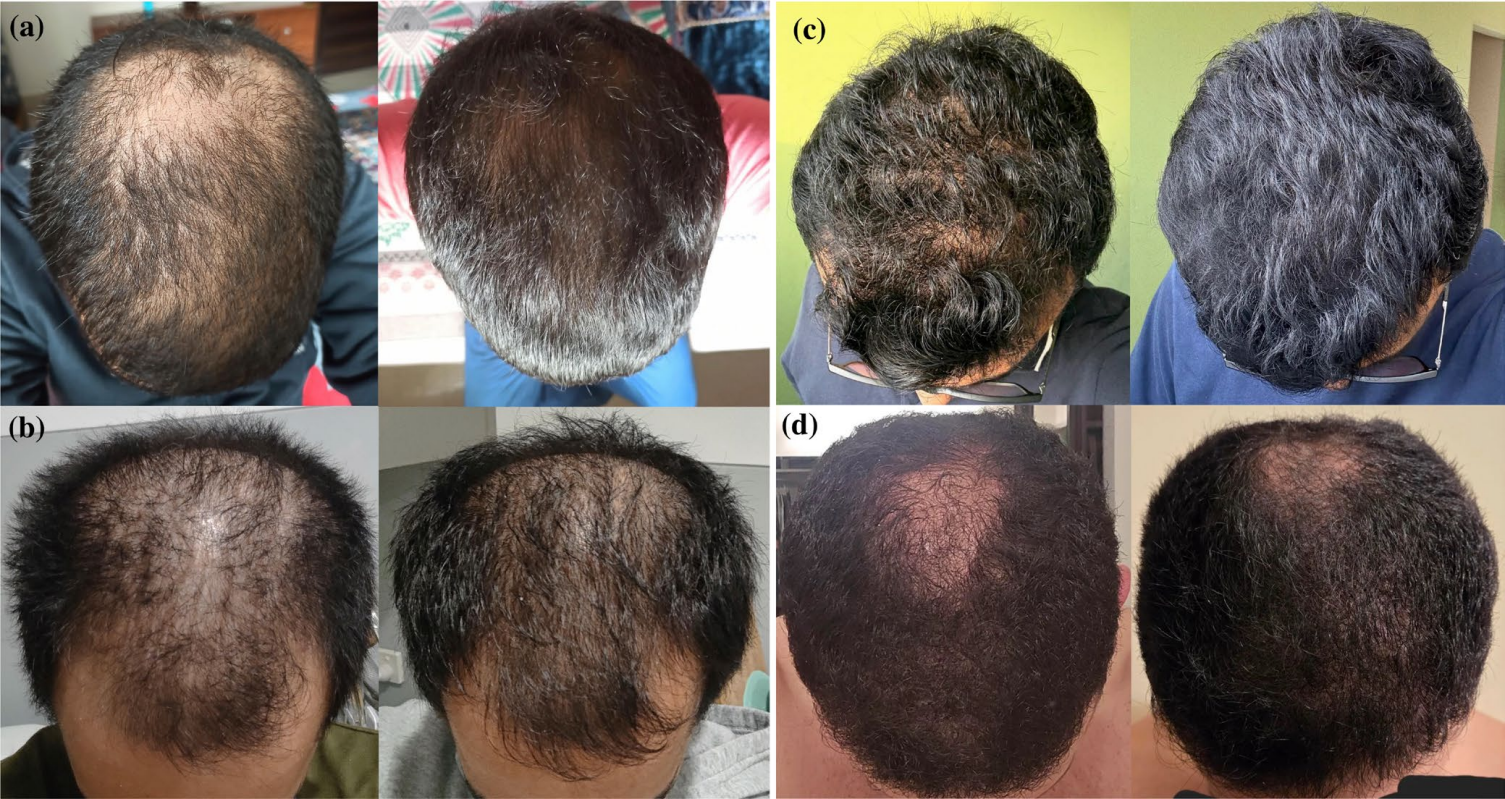
Representative clinical photographs of male patients with AGA in two groups before and after treatment. (a) and (b) are patients treated with topical minoxidil before treatment (left) and after 6 months of treatment (right). (c) and (d) are patients treated with oral minoxidil before treatment (left) and after 6 months of treatment (right).

**FIGURE 6:**
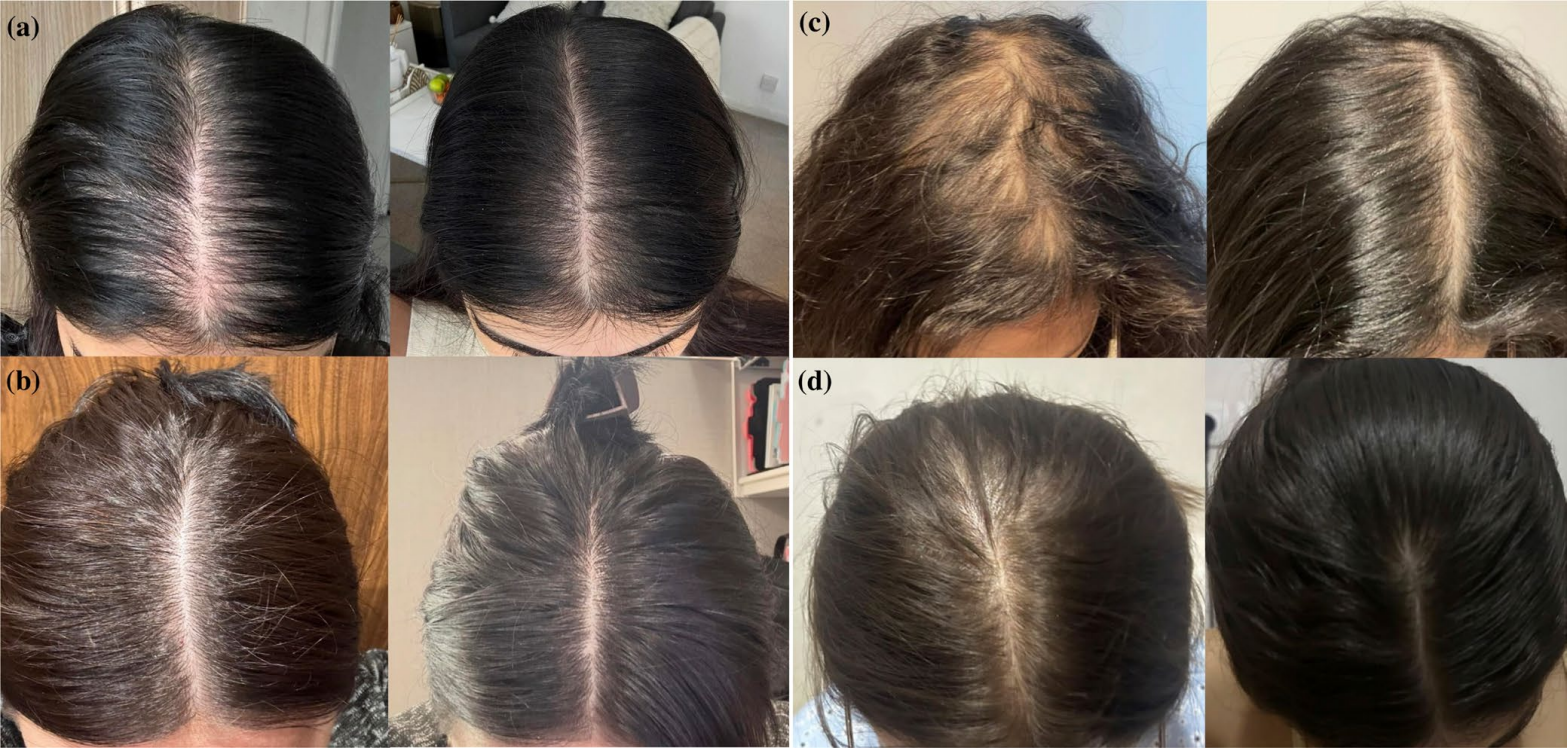
Representative clinical photographs of female patients with AGA in two groups before and after treatment. (a) and (b) are patients treated with topical minoxidil before treatment (left) and after 6 months of treatment (right). (c) and (d) are patients treated with oral minoxidil before treatment (left) and after 6 months of treatment (right).

Wilcoxon signed‐rank test comparing change scores to zero (no change). Significant *p*‐values indicate improvement from baseline and do not imply differences between groups. Figures 5 and 6 represent the clinical photographs of patients at baseline and after 24 weeks of treatment with oral and topical minoxidil respectively.

Table 6 summarizes the adverse effects observed, the most common being hypertrichosis, which was significantly more frequent in the oral group compared to topical (14 [30%] vs. 4 [9.1%], *p* = 0.011). In both groups, hypertrichosis was generally well‐tolerated. Other skin‐related complications, such as contact dermatitis (11.4% vs. 0%, *p* = 0.025) and seborrheic dermatitis (6.8% vs. 0%), were more common with topical minoxidil, whereas systemic cardiovascular effects, like pedal edema (4.3%) and tachycardia (8.7%) occurred in the oral minoxidil group. Four patients in the oral group reported orthostatic hypotension, but it was mild and controlled through increasing salt intake, and all patients continued the study. Early telogen effluvium was observed in 6.5% and 9.1% of oral and topical minoxidil patients, respectively, with no differences in resting heart rate.

**TABLE 6:** Adverse effects by treatment group.

Adverse effects	Topical *N* (%)	Oral *N* (%)	Risk difference (95% CI)	*p*
Hypertrichosis	4 (9.1%)	14 (30.4%)	+20.9% (+5.8% to +36.0%)	0.011
Contact dermatitis	5 (11.4%)	0 (0.0%)	−11.4% (−21.6% to −1.2%)	0.025
Seborrheic dermatitis	3 (6.8%)	0 (0.0%)	−6.8% (−15.1% to +1.5%)	0.113
Telogen effluvium	4 (9.1%)	3 (6.5%)	−2.6% (−13% to +8%)	0.711
Orthostatic hypotension	1 (2.3%)	4 (8.7%)	+6.4% (−3.0% to +15.8%)	0.361
Pitting edema	0 (0.0%)	2 (4.3%)	+4.3% (−2.5% to +11.1%)	0.495
Tachycardia	0 (0.0%)	4 (8.7%)	+8.7% (−2.5% to +19.9%)	0.117
Headache/light‐headedness	2 (4.5%)	2 (4.3%)	−0.2% (−8% to +8%)	1.000

*Note:* Risk difference calculated as Oral − Topical. Confidence intervals approximate based on binomial proportions.

Throughout the 6‐month study, no serious adverse events were observed. The change in blood pressure over 24 weeks was comparable between groups (ΔSBP: *p* = 0.669; ΔDBP: *p* = 0.948), indicating no clinically meaningful cardiovascular effect of either treatment (Table 3).

## 4 Discussion

In this randomized, double‐blind, double‐dummy controlled trial, low‐dose oral minoxidil demonstrated outcomes comparable to topical minoxidil in the treatment of androgenetic alopecia over 24 weeks. Clinician‐assessed improvement (≥ +1) was observed in 67.4% of patients receiving oral therapy compared with 72.7% receiving topical therapy, corresponding to an absolute difference of −5.3% (95% CI: −22.0% to +12.0%). This was mirrored by identical median Dermatologist Assessment Scores, with a difference of 0.0 (95% CI: −1.0 to +1.0), supporting comparable observed efficacy. The confidence interval spans both potential benefit and harm, indicating uncertainty regarding any true difference between treatments.

Our secondary finding revealed a consistent numerical trend favoring oral minoxidil in patient‐reported satisfaction, with a +9.9% higher response rate (95% CI: −6.5% to +26.3%) and a median difference of +1.0 (95% CI: 0.0 to +1.0). This widened across higher thresholds (≥ +2 difference: +20.1%; 95% CI: −1.5% to +41.7%), though without formal statistical significance. In contrast, clinician‐assessed outcomes were closely aligned across all thresholds, with small differences centered around zero and wide confidence intervals.

Taken together, these findings suggest that any true difference in efficacy between oral and topical minoxidil is likely modest within the timeframe studied. Because this study was powered to detect a predefined difference rather than non‐inferiority, the absence of a significant difference should not be interpreted as proof of therapeutic equivalence, but rather as indicating that clinically meaningful differences, if present, could not be reliably detected within the available sample size.

The observed divergence between clinician‐assessed outcomes and patient‐reported satisfaction is noteworthy. While objective improvement appeared similar between groups, patient‐perceived benefit tended to favor oral therapy. This may reflect factors such as avoidance of topical application burden, once‐daily dosing, and lifestyle restriction—such as a 4‐h post‐application window refraining from swimming or shampooing—which are known to influence adherence and patient preference. Given that adherence was not directly quantified using objective measures, its contribution to treatment response cannot be fully determined.

Our results align with a growing body of comparative evidence [12]. Ramos et al. [13] compared 1 mg oral minoxidil with 5% topical solution in 52 women, with the former producing a numerically greater but statistically insignificant increase in total hair density (12% vs. 7.2%; *p* = 0.09). Our study advances this insight by demonstrating that even lower‐strength TM (2% in females) achieves short‐term efficacy comparable to low‐dose OM. Similarly, Vahabi‐Amlashi et al. demonstrated no significant intergroup differences in hair density or diameter when comparing oral (0.25 mg) with topical minoxidil (2%) in 72 women over 9 months, despite significant improvements from baseline in both arms [14]. Their work suggests effective hair growth can be achieved at oral doses lower than our regimen (0.25 vs. 1.25 mg). Penha et al. [15] compared 5 mg oral minoxidil, a higher dose than used in our study. Photographic assessment, however, demonstrated the superiority of oral minoxidil at the vertex (24%; *p* = 0.04), but not at the frontal scalp. In Asilian et al. [16] 1 mg oral minoxidil matched 5% topical solution's efficacy over 6 months, with photographic assessments favoring topical for density gains, while oral showed non‐significant changes. Patient satisfaction was higher in the topical group (76% vs. 67%), opposite to our trend, possibly due to the lower 1 mg dose used in their study. Ultimately, a recent meta‐analysis of randomized clinical trials found no statistically significant differences in hair density or diameter between oral and topical minoxidil, anchoring our findings within the consensus [17].

The safety profiles of the two formulations were distinct but generally consistent with prior literature [18]. Hypertrichosis occurred more frequently with oral minoxidil, with an absolute risk increase of +20.9% (95% CI: +5.8% to +36.0%), matching trends identified by Ramos et al. (27% vs. 4%) [13] and Penha et al. (49% vs. 25%; *p* = 0.02) [15]. In contrast, topical therapy was associated with a higher incidence of localized dermatologic adverse effects, including contact dermatitis (+11.4%, 95% CI: +1.2% to +21.6%). Penha noted that scalp eczema (16% versus 2%, *p* = 0.02) and shedding (16% versus 9%) were more common in the topical minoxidil group, and headache was more common in the oral group (14% versus 2%) [15]. Systemic adverse effects in the oral group, including tachycardia and orthostatic hypotension, were infrequent and mild, with confidence intervals encompassing small absolute risks. This may be explained by the fact that the serum concentration threshold for cardiac effects is 20 ng/mL, while its hair growth effects can be achieved at levels above 1.6 ng/mL. At a dose of 2.5 mg, oral minoxidil reaches peak plasma levels within 1 h, staying below the cardiovascular threshold while remaining above the hair growth threshold for 4 h [19]. However, the sample size and duration were insufficient to exclude rare or delayed adverse events.

Several limitations should be considered. The study was not designed as a non‐inferiority or equivalence trial, and the confidence intervals around treatment effects were relatively wide, limiting the precision of comparative estimates. In addition, while outcomes were measured using validated global photographic assessment scales, they are inherently subjective and may lack sensitivity to detect subtle differences. The absence of objective quantitative measures, such as phototrichogram‐derived hair density and shaft diameter, limits precision. Lastly, the 24‐week duration may not capture long‐term treatment effects or the sustainability of response in this chronic condition, whereas the single‐centre design may limit generalizability and introduce potential reporting bias.

Despite these limitations, the study has important strengths, including its randomized, double‐blind, double‐dummy design, use of sex‐specific dosing regimens, and detailed safety assessment. The consistency of findings across multiple outcome measures and analytic approaches supports the robustness of the observed results within the constraints of the study.

In clinical practice, the choice between oral and topical minoxidil should consider not only efficacy but also patient preference, tolerability, and adherence. The present findings suggest that both formulations offer similar short‐term benefits, with distinct adverse effect profiles that may guide individualized treatment decisions.

## 5 Conclusion

In this randomized controlled trial, low‐dose oral minoxidil and topical minoxidil both produced clinically meaningful improvements in androgenetic alopecia over 24 weeks, with no statistically significant differences in efficacy between treatment groups. While patient satisfaction showed a non‐significant trend favoring oral therapy, clinician‐assessed outcomes were comparable.

These findings suggest that low‐dose oral minoxidil may serve as a practical alternative to topical therapy in appropriately selected patients, particularly those with adherence challenges or intolerance to topical formulations. However, given that this study was not designed to establish non‐inferiority or equivalence, the results should be interpreted as indicating comparable observed outcomes rather than definitive therapeutic equivalence.

Both treatments demonstrated favorable safety profiles, with distinct but generally well‐tolerated adverse effect patterns. Larger, multicenter studies with longer follow‐up durations and objective outcome measures are needed to further define the comparative effectiveness, long‐term safety, and optimal patient selection for oral versus topical minoxidil in androgenetic alopecia.

## Author Contributions

Muhammad Asad Ejaz Chaudhry, Bushra Muneeb, and Safoora Aamir contributed to conception, design, and methodology. Muhammad Asad Ejaz Chaudhry and Bushra Muneeb contributed to the formal analysis and interpretation of data for the work. Muhammad Asad Ejaz Chaudhry and Zoha Arif Saeed contributed to the investigation and data acquisition. Muhammad Asad Ejaz Chaudhry, Sidra Ashraf, and Zoha Arif Saeed contributed to writing and editing the paper. Shoaib Ashraf contributed to funding acquisition and resources. Safoora Aamir and Shoaib Ashraf supervised the study. All authors read and agree with the final approval of the version to be published.

## Funding

The authors have nothing to report.

## Disclosure

No medical writing, editorial, or other assistance was received in the preparation of this manuscript. Patients were not involved in the design, conduct, reporting, or dissemination of this research.

## Ethics Statement

This study was approved by the Institutional Review Board of Shaikh Zayed Hospital (Ref. No.: TERC/SC/INT/2025/363) on 17/02/2025. Written informed consent was obtained from all participants before enrollment. Additional written consent for the publication of clinical photographs was obtained from all patients depicted. The study was conducted in accordance with the Declaration of Helsinki.

## Conflicts of Interest

The authors declare no conflicts of interest.

## Data Availability

The datasets generated and analyzed during the current study are available from the corresponding author on reasonable request.
